# Physical match performance of elite soccer players from the English Championship League and the English Premier League: The effects of opponent ranking and positional differences

**DOI:** 10.5114/biolsport.2025.139079

**Published:** 2024-05-07

**Authors:** Ryland Morgans, Rocco Di Michele, Ibrahim H. Ceylan, Ben Ryan, Chris Haslam, Matthew King, Piotr Zmijewski, Rafael Oliveira

**Affiliations:** 1 School of Sport & Health Sciences, Cardiff Metropolitan University, Cardiff, UK; 2 Department for Life Quality Studies, University of Bologna, Bologna, Italy; 3 Faculty of Kazim Karabekir Education, Physical Education of Sports Teaching Department, Ataturk University, Erzurum, Turkey; 4 Football Research Centre, Brentford FC, London, UK; 5 Jozef Pilsudski University of Physical Education in Warsaw, 00-809 Warsaw, Poland; 6 Research and Development Center Legia Lab, Legia Warszawa, Poland; 7 Research Centre in Sports Sciences, Health and Human Development, 5001–801 Vila Real, Portugal; 8 Sports Science School of Rio Maior – Instituto Politecnico de Santarem, 2040–413 Rio Maior, Santarém District, Santarém, Portugal; 9 Life Quality Research Centre, 2040–413 Rio Maior, Portugal

**Keywords:** Soccer, Match analysis, Physical performance, Seasonal trends, Standard of competition, Opponent ranking, Positional differences

## Abstract

This study aimed to examine physical match performance and the effects of opponent ranking and positional differences in both the English Championship League (ECL) and the English Premier League (EPL) over five consecutive seasons. Fifty-four professional outfield soccer players (average age 24.6 ± 5.4 years, weight 76.6 ± 6.9 kg, height 1.79 ± 0.09 m) from an English club were involved. Physical data obtained with the 18 Hz GPS technology tracking system from 213 regular-season matches spanning the complete 2018/19 to 2022/23 seasons were examined. The results showed that, considering the level of the opponent, total distance (TD), high-intensity distance (HSR), and the number of decelerations (DEC) significantly varied in both EPL and ECL (p < 0.05, p < 0.05, p < 0.05 respectively). Additionally, opponent level influenced sprint distances (Spr) and accelerations (ACC) in the EPL (p < 0.05, p < 0.05) but not in the ECL. The highest running metrics were noted when the team played against a high-ranked opponent. Concerning positional roles, more physical metrics were influenced by opponent level in the ECL (centre-backs (TD, ACC), full-backs (TD, DEC), centre midfielders (TD, HSR, Spr, DEC), attacking midfielders (TD, Spr, DEC), centre forwards (TD)) than in the EPL (centre midfielders (TD, HSR, DEC), attacking midfielders (TD, DEC), centre forwards (TD)). These findings contribute to a more comprehensive understanding of how players from different positions perform in elite soccer match-play against varying opposition rankings. Coaches may then tailor tactical approaches, positional, and individualized training regimens to address the specific physical demands associated with matches against different-ranked opponents.

## INTRODUCTION

Elite soccer demands a multifaceted understanding of the various factors influencing player performance, with opponent ranking, league status and positional differences emerging as some examples of crucial determinants [1]. The relationship between opponent ranking and player performance has been a subject of consistent exploration. Recent research has highlighted the heightened physical demands imposed by high-ranked opponents [2]. These findings highlight the need for players and teams to adapt to the varying challenges posed by quality opponents. Understanding the fluctuations in physical outputs across different levels of opposition quality (low-, middle- and high-ranked teams) is pivotal for devising effective training and tactical strategies [3].

Differing positional functions have a critical role in shaping the physical demands placed on players during matches. The distinct physical profiles associated with different playing positions have recently been examined [4–8]. For instance, some studies showed that central midfielders covered higher total distance (TD) at low and medium intensities, as well as moderate-intensity acceleration (ACC) distances when compared to attackers and defenders, among elite English Premier League (EPL) [4] and Spanish First Division [6] soccer players. Additionally, it was verified that wide attackers and wide defenders have shown the highest values of very high-speed running, high-intensity ACC, and sprint distances (Spr) due to the perpetual attacking and defensive functions in the EPL [4]. Lastly, in a study that assessed the position-specific development of physical performance parameters over a seven-season period in the EPL, it was found that wide and forward positions increased high-intensity distance (HSR) and Spr more than central defenders and central midfielders [9]. Lastly, a recent study showed that there were significant increases (from 2014/15 to 2018/19 season) in: TD for all position with the exception of defensive midfielders, attacking midfielders and wide midfielders; HSR distance for all positions and Spr distance for all positions except central midfielders and attacking midfielders [10].

Understanding how these positional differences interact with the challenges posed by varying levels of opposition ranking is essential for tailoring training programs and optimizing team strategies [11] to cope with these identified demands. This is even more relevant when working within the same teams for several years in which the team could go though different participating levels (e.g., playing in a European competition, to being relegated and playing in differen divisions). The ability of players and teams to adapt to the diverse challenges posed by both opponent ranking and positional differences is a recurrent theme in the literature [8]. However, to the best of the authors’ knowledge, there is currently no existing literature characterizing the physical match performance between the English Championship League (ECL) and the EPL in relation to opponent ranking and the effects on positional demands across five seasons.

The concept of evaluating physical performances over an extended duration, as proposed in the present study, aligns with a growing trend in soccer research [7, 8]. Longitudinal studies emphasize the importance of analyzing performance trends over multiple seasons [7–9]. This approach enables a nuanced understanding of how players adapt, evolve, and maintain physical output over time, contributing significantly to the literature on player development.

The capacity to analyze the impact of opponent ranking and league status on differences in physical match performance of the same team participating in different league standards holds practical significance and would contribute to our understanding of specific positional distinctions between the ECL and EPL competition. Therefore, the aim of this study was to examine physical match performance and the effects of opponent ranking and positional differences with-in both the ECL and the EPL over five consecutive seasons. Based on recent research, it was hypothesized that opponent ranking and playing positions would affect physical match performance in both the ECL and EPL.

## MATERIALS AND METHODS

### Study Design

This research utilized a five-year longitudinal study design. A nonprobabilistic sampling protocol was employed to recruit the participants. The emphasis of the study was on monitoring player load and the effects of opponent ranking and positional differences within both the ECL and the EPL during competitive matches. During the observation period of seasons 2018/19 to 2022/23, consistent player monitoring approaches were implemented without any interference from the researchers [12].

### Participants

Fifty-four professional outfield soccer players from an English club were involved in the study. Data from the complete 2018/19 to 2022/23 seasons included 54 senior players (first-team squad) (age 24.6 ± 5.4 years, weight 76.6 ± 6.9 kg, height 1.79 ± 0.09 m). The research inclusion criteria have been previously applied [8] and were: (i) named in the first-team squad at the start of all study seasons, (ii) played in at least 80% of matches, and (iii) only completed official team training during the study period. Additionally, the exclusion criteria for the study have also been previously employed [8] and included: (i) long-term (three months or longer) injured player data, (ii) joining the team late in either of the study seasons, (iii) lack of full, complete match data, (iv) an in-sufficient number of satellite connection signals, and (v) goalkeepers, due to the different variations in the physical demands with outfield players [13].

Players were classified into one of five positions due to varying match demands. These were: centre-backs (CB; n = 13), full-backs (FB; n = 7), centre midfielders (CM; n = 19), attacking midfielders (AM; n = 15), and centre forwards (CF; n = 6). All data collected resulted from normal analytical procedures regarding player monitoring over the competitive season [8], nevertheless, written informed consent was obtained from all participants. The study was conducted according to the requirements of the Declaration of Helsinki and was approved by the local Ethics Committee of Cardiff Metropolitan University and the club from which the participants volunteered [14]. To ensure confidentiality, all data were anonymized prior to analysis.

### Data Collection

Data were collected in all (n = 213) regular-season matches played by the examined team across the five study seasons. In the 2018/19 season, one scheduled regular-season match for the team was forfeited and thus not played. The team participated in the ECL for three out of the examined seasons (2018/19 to 2020/21), playing a total of n = 137 regular-season matches, and in the EPL for two seasons (2021/22 and 2022/23), playing a total of n = 76 regular-season matches.

Physical match data were consistently monitored across the study seasons using an 18 Hz Global Positioning System (GPS) technology tracking system (Apex Pod, version 4.03, 50 gr, 88 × 33 mm; Statsports; Northern Ireland, UK) that has been previously validated for tracking distance covered and peak velocity during simulated team sports and linear sprinting [15]. All devices were activated 30-minutes before data collection to allow the acquisition of satellite signals and to synchronize the GPS clock with the satellite’s atomic clock [16]. All data collection procedures and unit error and reliability have previously been reported [16–19]. To avoid inter-unit error, each player wore the same device during the study period [20].

Following every match, running data were extracted and processed prior to analysis using proprietary software (Apex, 10 Hz version 4.3.8, Statsports Software; Northern Ireland, UK) as softwarederived data is a more simple and efficient way for practitioners to obtain data in an applied environment, with no differences reported between processing methods (software-derived to raw processed) [20]. The minimum effort duration (dwell time) to quantify HSR (0.5 s) and Spr (1 s) have previously been suggested [20]. Additionally, to display a higher level of precision and less error, the GPS internal processing utilized the Doppler shift method to calculate both distance and velocity [21].

Variables analyzed have previous validated in elite soccer [22, 23], and were divided by actual playing time for each player. The absolute TD covered (m/min); HSR (m/min, distance covered 5.5–7 m·s^−1^); Spr (m/min, distance covered > 7 m · s^−1^) were examined. The following physical variables were also quantified in this study: the number of accelerations (ACC) (> +3 m · s^−2^ with minimum duration of 0.5 s); and the number of decelerations (DEC) (< -3 m · s^−2^ with minimum duration of 0.5 s) [24, 25].

For the subsequent analyzes, all individual recordings with playing time of 15-minutes or more [26] were examined (n = 2632, with an average of 12.4 data points per match). Every match was classified according to the opponent team ranking, determined according to the final position in the previous season [27]. For the ECL seasons, teams were ranked as high-ranked (the three teams relegated from the EPL, and the three teams placed in positions three to six in the ECL not promoted to the EPL), middle-ranked (seventh to fifteenth), and low-ranked (sixteenth to twenty-first and the three teams promoted from the English Football League One). For the EPL seasons, teams were ranked as high-ranked (first to sixth), middle-ranked (seventh to fifteenth) and low-ranked (sixteenth, seventeenth, and the three teams promoted from ECL).

### Statistical analysis

All data are presented as the mean ± standard deviation (SD). Linear mixed-effect models, with random intercepts on individual players IDs, were used to assess the effect of opponent team ranking and players position on the examined physical performance metrics. Tukey’s pairwise comparisons were performed to assess the differences between opposition levels within each playing position. The analysis was conducted separately for the ECL and EPL periods. The estimated differences were divided by the estimated between-player standard deviations to calculate the effect size (ES). The absolute ES values were evaluated as < 0.2 trivial, 0.2–0.6 small, 0.6–1.2 moderate, 1.2–2.0 large, 2.0–4.0 very large, and > 4.0 extremely large [28]. The analyzes were carried out with the software R Software, version 4.3.1 (R Development Core Team, Vienna, Austria). Statistical significance was set at p < 0.05.

## RESULTS

### English Championship League

**FIG. 1:**
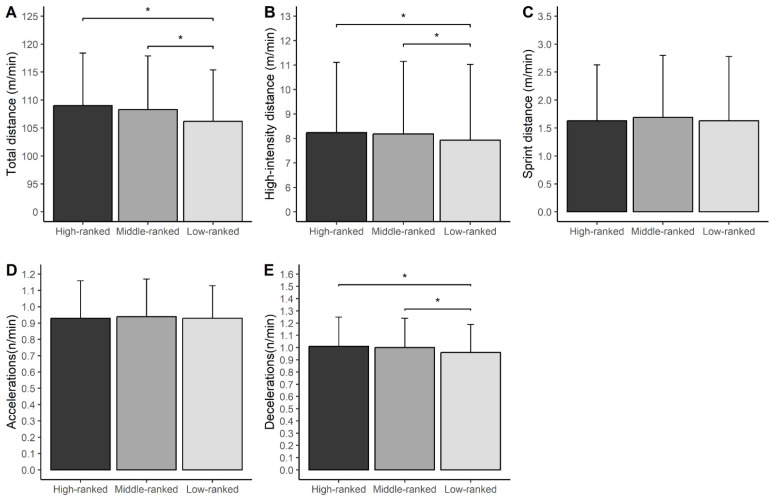
Mean and SD values for total distance (A); high-intensity distance (B); sprint distance (C); number of accelerations (D); and number of decelerations (E) in the ECL period (seasons 2018/19 to 2020/21) in relation to opponent ranking. * p < 0.05.

Figure 1 shows the mean values of the examined running metrics across the 2018/19 to 2020/21 seasons (ECL period) irrespective of playing position.

The relative TD covered (Figure 1A), the HSR (Figure 1B), and the number of decelerations (Figure 1E) were lower in matches played against low-ranked opponents vs. matches played against both high- and middle-ranked opponents. The effect sizes were small for the relative TD covered (ES = 0.35 for matches played against low-ranked vs. high-ranked opponents; ES = 0.26 for matches played against low-ranked vs. middle-ranked opponents), and the number of decelerations (ES = 0.33 for matches played against low-ranked vs. high-ranked opponents; ES = 0.27 for matches played against low-ranked vs. middle-ranked opponents), while they were trivial for the HSR (ES = 0.19 for matches played against low-ranked vs. high-ranked opponents; ES = 0.14 for matches played against low-ranked vs. middle-ranked opponents). No significant differences between opposition rankings were observed for Spr (Figure 1C) and number of ACC (Figure 1D).

Table 1 compares all opposition levels of all variables for the ECL team considering the different playing positions.

**TABLE 1:** Mean ± SD values for the examined physical performance metrics, aggregated by opposition ranking and playing positions, in the ECL period (seasons 2018/19 to 2020/21).

	High-ranked	Middle-ranked	Low-ranked
**CENTRE-BACKS**			
Total distance (m/min)	98.98 ± 6.19 ^#^	98.61 ± 5.56^°^	96.98 ± 5.16
High-intensity distance (m/min)	5.61 ± 1.75	5.22 ± 1.39	5.25 ± 1.43
Sprint distance (m/min)	1.14 ± 0.67	0.97 ± 0.53	1.00 ± 0.53
Accelerations (n/min)	0.84 ± 0.18 ^*^	0.89 ± 0.21	0.89 ± 0.20
Decelerations (n/min)	0.79 ± 0.13	0.80 ± 0.16	0.77 ± 0.14

**FULL-BACKS**			
Total distance (m/min)	106.69 ± 5.89 ^#^	105.98 ± 5.45	104.28 ± 6.79
High-intensity distance (m/min)	8.03 ± 2.15	7.94 ± 1.99	7.77 ± 2.15
Sprint distance (m/min)	1.86 ± 0.74	1.87 ± 0.93	1.92 ± 0.95
Accelerations (n/min)	0.93 ± 0.14	0.96 ± 0.15	0.93 ± 0.13
Decelerations (n/min)	1.07 ± 0.16 ^#^	1.07 ± 0.16 ^°^	1.00 ± 0.15

**CENTRE MIDFIELDERS**			
Total distance (m/min)	116.29 ± 7.77 ^#^	114.66 ± 9.31 ^°^	111.94 ± 9.03
High-intensity distance (m/min)	8.52 ± 2.85 ^*^	8.09 ± 2.74 ^°^	7.39 ± 2.82
Sprint distance (m/min)	1.37 ± 1.00 ^#^	1.29 ± 0.87	1.16 ± 0.93
Accelerations (n/min)	0.92 ± 0.22	0.93 ± 0.22	0.90 ± 0.20
Decelerations (n/min)	1.05 ± 0.20 ^#^	1.03 ± 0.20 ^°^	0.97 ± 0.19

**ATTACKING MIDFIELDERS**			
Total distance (m/min)	111.04 ± 6.22 ^#^	110.83 ± 7.04^°^	109.16 ± 6.37
High-intensity distance (m/min)	10.28 ± 2.58	10.70 ± 2.59	10.62 ± 3.01
Sprint distance (m/min)	2.22 ± 1.12 ^§^	2.62 ± 1.19	2.43 ± 1.30
Accelerations (n/min)	1.00 ± 0.31	0.96 ± 0.30	0.97 ± 0.26
Decelerations (n/min)	1.08 ± 0.33 ^#^	1.04 ± 0.32	1.03 ± 0.32

**CENTRE FORWARDS**			
Total distance (m/min)	106.69 ± 8.50 ^#^	106.09 ± 8.28	104.66 ± 8.15
High-intensity distance (m/min)	8.84 ± 2.42	9.47 ± 2.64	9.22 ± 2.71
Sprint distance (m/min)	1.88 ± 0.96	2.16 ± 1.14	2.08 ± 1.21
Accelerations (n/min)	1.01 ± 0.18	1.01 ± 0.19	1.00 ± 0.16
Decelerations (n/min)	1.11 ± 0.17	1.11 ± 0.20	1.08 ± 0.19

* significant difference vs. middle-ranked and low-ranked (p < 0.05);

# significant difference vs. low-ranked (p < 0.05);

§ significant difference vs. middle-ranked (p < 0.05);

° significant difference vs. low-ranked (p < 0.05)

For centre-backs, the TD covered was lower against low-ranked opponents compared to high-(ES = 0.29, small) and middle-ranked opponents (ES = 0.23, small), whereas the number of ACC was higher when playing against low-(ES = 0.46, small) and middle-(ES = 0.35, small) compared to high-ranked opponents. For full-backs, the TD covered was greater against high-vs. low-ranked opponents (ES = 0.30, small). Also, the number of DEC was lower when playing against low-ranked opponents compared to both high-(ES = 0.38, small) and middle-ranked opponents (ES = 0.37, small). Centre midfielders showed greater values of HSR when playing against high-ranked vs. both middle-(ES = 0.22, small) and low-ranked opponents (ES = 0.53, small), and in matches against middle-ranked vs. low-ranked opponents (ES = 0.31, small). Also, centre midfielders showed lower TD and number of DEC when playing against low-ranked opponents compared to playing against both middle-(ES = 0.33, small, for TD; ES = 0.39, small, for number of DEC) and high-ranked opponents (ES = 0.52, small, for TD; ES = 0.51, small, for number of DEC), and lower Spr against low-vs. high-ranked opponents (ES = 0.33, small). Attacking midfielders covered lower TD when playing against low-ranked opponents, compared to both high-(ES = 0.26, small) and middle-ranked opponents (ES = 0.24, small). Furthermore, this position performed a higher number of DEC in matches against high-ranked teams vs. matches against low-ranked teams (ES = 0.38, small). Attacking midfielders also produced lower Spr in matches against high-ranked teams compared to middle-ranked opponents (ES = 0.50, small). Finally, centre forwards covered greater TD in matches against high-ranked teams as opposed to matches against low-ranked teams (ES = 0.29, small).

### English Premier League

**FIG. 2:**
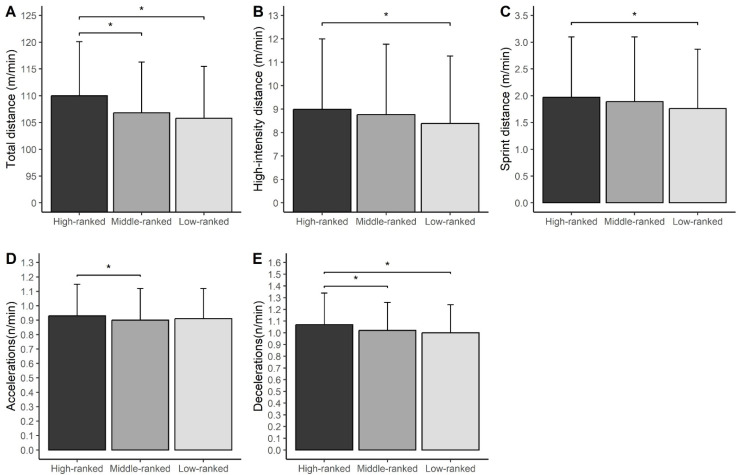
Mean and SD values for total distance (A); high-intensity distance (B); sprint distance (C); number of accelerations (D); and number of decelerations (E) in relation to opponent ranking in the EPL period (seasons 2021/22 and 2022/23). * p < 0.05

Figure 2 shows the mean values of the examined metrics across the 2021/22 and 2022/23 seasons (EPL period) irrespective of playing position.

The TD completed was greater in matches played against high-ranked opponents compared to those played against both middle-(ES = 0.50, small) and low-ranked opponents (ES = 0.64, moderate) (Figure 2A). The HSR (Figure 2B) and Spr (Figure 2C) were greater in matches played against high-ranked teams vs. those played against low-ranked teams (ES = 0.32, small for HSR; ES = 0.31, small, for Spr). The number of ACC was higher in matches against high-ranked opponents vs. those played against middle-ranked opponents (ES = 0.17, trivial) (Figure 2D), where-as the number of DEC was higher in matches played against high-ranked opponents compared to those played against both middle-(ES = 0.26, small) and low-ranked opponents (ES = 0.35, small) (Figure 2E).

Table 2 compares all opposition levels of all variables for the EPL team considering the different playing positions.

**TABLE 2:** Mean ± SD values for the examined physical performance parameters, aggregated by opposition ranking and playing positions, in the EPL period (seasons 2021/22 and 2022/23).

	High-ranked	Middle-ranked	Low-ranked
**CENTRE-BACKS**			
Total distance (m/min)	100.07 ± 6.92	98.92 ± 7.23	98.59 ± 7.87
High-intensity distance (m/min)	6.05 ± 1.87	5.67 ± 1.70	6.08 ± 1.99
Sprint distance (m/min)	1.35 ± 0.74	1.18 ± 0.73	1.27 ± 0.83
Accelerations (n/min)	0.79 ± 0.15	0.77 ± 0.15	0.81 ± 0.13
Decelerations (n/min)	0.83 ± 0.14	0.80 ± 0.14	0.79 ± 0.16

**FULL-BACKS**			
Total distance (m/min)	107.81 ± 7.13	104.40 ± 5.60	105.03 ± 7.59
High-intensity distance (m/min)	9.50 ± 2.46	8.97 ± 1.75	9.00 ± 2.37
Sprint distance (m/min)	2.58 ± 1.14	2.19 ± 0.91	2.27 ± 1.11
Accelerations (n/min)	0.97 ± 0.17	0.95 ± 0.17	0.96 ± 0.17
Decelerations (n/min)	1.20 ± 0.21	1.14 ± 0.19	1.13 ± 0.18

**CENTRE MIDFIELDERS**			
Total distance (m/min)	117.31 ± 7.64 ^*^	113.60 ± 8.02 ^°^	111.17 ± 9.13
High-intensity distance (m/min)	9.94 ± 2.66 ^#^	9.54 ± 2.78 ^°^	8.68 ± 2.9
Sprint distance (m/min)	1.74 ± 1.02	1.68 ± 1.05	1.47 ± 1.03
Accelerations (n/min)	0.93 ± 0.22	0.88 ± 0.20	0.88 ± 0.20
Decelerations (n/min)	1.18 ± 0.26 ^*^	1.09 ± 0.21	1.04 ± 0.22

**ATTACKING MIDFIELDERS**			
Total distance (m/min)	113.97 ± 7.48 ^*^	107.59 ± 7.24	107.37 ± 6.28
High-intensity distance (m/min)	11.12 ± 2.49	10.96 ± 2.65	10.40 ± 2.36
Sprint distance (m/min)	2.85 ± 1.14	2.96 ± 1.41	2.68 ± 1.06
Accelerations (n/min)	1.09 ± 0.27	1.03 ± 0.27	1.04 ± 0.28
Decelerations (n/min)	1.15 ± 0.27 ^*^	1.07 ± 0.26	1.07 ± 0.28

**CENTRE FORWARDS**			
Total distance (m/min)	102.09 ± 5.76 ^*^	97.65 ± 5.69	97.62 ± 9.39
High-intensity distance (m/min)	7.59 ± 1.78	7.06 ± 1.89	7.21 ± 2.15
Sprint distance (m/min)	1.61 ± 0.63	1.46 ± 0.63	1.35 ± 0.55
Accelerations (n/min)	0.95 ± 0.09	0.87 ± 0.10	0.81 ± 0.12
Decelerations (n/min)	0.91 ± 0.13	0.88 ± 0.12	0.92 ± 0.19

* significant difference vs. middle-ranked and low-ranked (p < 0.05);

# significant difference vs. low-ranked (p < 0.05);

° significant difference vs. low-ranked (p < 0.05)

Centre-backs and full-backs showed no significant differences between opposition rankings for any of the examined variables. Centre midfielders showed greater TD covered in matches played against high-ranked opponents compared with both middle-(ES = 0.46, small) and low-ranked opponents (ES = 0.80, moderate), and in matches played against middle-ranked opponents vs. those played against low-ranked opponents (ES = 0.34, small). Centre midfielders also covered lower HSR in matches against low-ranked opponents compared to matches against both high-(ES = 0.51, small) and middle-ranked opponents (ES = 0.37, small) and performed a higher number of DEC in matches against high-ranked vs. both low-(ES = 0.53, small) and middle-ranked opponents (ES = 0.33, small). Attacking midfielders covered greater TD and a higher number of DEC in matches against high-ranked opponents compared to matches against both middle-(ES = 0.84, moderate for TD; ES = 0.26, small for number of DEC) and low-ranked opponents (ES = 0.91 moderate for TD; ES = 0.35, small for number of DEC). Finally, centre forwards covered greater TD in matches against high-ranked opponents compared to matches against both middle-(ES = 0.49, small) and low-ranked opponents (ES = 0.72, moderate) (Table 2).

## DISCUSSION

The aim of this study was to examine physical match performance and the effects of opponent ranking and positional differences in both the ECL and the EPL over five consecutive seasons using data from one club.

The main observations from this study in the ECL were that TD covered, HSR and the number of DEC were significantly lower in matches played against low-ranked opponents. For centre-backs, the TD covered was lower against low-ranked opponents, whereas the number of ACC was lower when playing high-ranked opponents. For full-backs, TD and the number of DEC was also lower when playing against low-ranked opponents. Centre midfielders showed greater values of TD, HSR and DEC, when playing against high-ranked opponents and produced higher Spr against high-ranked opponents. Attacking midfielders covered greater TD and performed a higher number of DEC in matches against high-ranked teams and produced lower Spr in matches against high-ranked teams. Finally, centre forwards covered greater TD in matches against high-ranked teams.

Additionally, in the EPL the TD, HSR and Spr and the number of ACC and DEC completed was greater in matches played against high-ranked opponents. Centre and attacking midfielders showed greater TD and a higher number of DEC in matches played against high-ranked opponents and covered lower HSR in matches against low-ranked opponents. Finally, centre forwards covered greater TD in matches against high-ranked opponents. For clarity, the discussion was divided into four sub-sections: ECL; EPL; practical applications; limitations and future research direction.

### English Championship League

Our findings reveal that total distance varied considering the level of opposition by teams. Matches played against both high- and middle-ranked opponents demonstrated a significantly greater TD covered compared to low-ranked opponents. This pattern aligns with the notion that higher-ranked opponents often present greater challenges, necessitating increased physical effort and work rate from teams to compete at that level [11, 12].

Interestingly, the analysis of HSR further refines the understanding of physical exertion patterns. Matches against low-ranked opponents were characterized by lower HSR when compared to matches against both high- and middle-ranked opponents. This finding may indicate a strategic adjustment in playing style when facing perceived weaker opposition, where the study team may have attempted a more controlled possession or tactical organization over high-intensity bursts of activity [2, 30]. Conversely, against higher ranked opponents, a greater emphasis on dynamic, high-intensity movements may be employed to exploit potential gaps or respond to the increased pace of the match while in possession or to an increase in TD and HSR when out of possession and chasing the ball.

The absence of significant differences in Spr and the number of ACC across different opposition rankings suggests that, irrespective of the opponent’s ranking, teams maintain a consistent frequency of ACC and very-high intensity events during match-play. This may be attributed to the inherent nature of soccer, where ACC and Spr are integral elements to offensive and defensive strategies [2] and may also be reflective of the examined individual player characteristics such as the ability to produce force rapidly. Still, this finding is not unique in the literature, as recent data, albeit from a lower standard of competition and age group, the English Premier Development League (U-23), highlighted similar findings [12]. Conversely, the number of DEC displayed a noteworthy pattern, with matches against low-ranked opponents exhibiting a lower volume than matches against both high- and middle-ranked opponents. The elevated number of DEC against higher-ranked opponents may signify the need for rapid changes in pace and direction, reflecting the heightened intensity and dynamic nature of matches against high-quality opponents [28] and potentially reflect lower possession thus producing more explosive, DEC actions when attempting to regain the ball which contrast with ACC actions. Such findings were also evident in a previous study conducted with Iranian professional soccer players [32]. Additionally, the greater number of sprints performed by top-level teams may also contribute to higher ACC and DEC due to differing tactical strategies [32]. Nonetheless, other situational factors such as match importance (stage of the season), intensity, score-line, competitive anxiety, higher level of athletes’ commitment, and the psychological pressure on the players from the top-level team may have contributed to the increased distance covered in these ACC and DEC zones [33]. An additional justification for the contrasting results between ACC and DEC may be associated with the fact that both actions need to occur with a minimum duration of 0.5 s. Thus, this may imply that ACC may have been performed quicker than DEC and not counting as an ACC action. Consequently, this may be a new topic of interest for future research.

The nuanced variations in physical performance metrics among different playing positions against high-, middle-, and low-ranked opponents provide valuable insights into the positional demands and strategic adaptations within the ECL. Specifically, for centre-backs, the observed greater TD covered against high-ranked opponents aligns with the expectation that matches against stronger opposition demand increased defensive efforts and greater involvement in play-making, necessitating more distance covered [3]. On the contrary, the higher number of ACC against low- and middle-ranked opponents suggests a proactive defensive approach, potentially involving quick changes in direction or pace in response to attacking situations against perceived weaker opponents. Notwithstanding, this finding was not corroborated by a recent study conducted in English Premier Development League (U-23) [12] in which higher values were found against high-ranked teams.

For full-backs the greater TD covered against high-ranked opponents echoes the demands of the dual-role, often requiring defensive resilience and offensive support [33]. However, the absence of significant differences in other metrics suggests that the overall demands placed on full-backs, irrespective of the opponent’s rank, may be more consistent than those of other positions examined.

The multifaceted role of centre midfielders is reflected in the varied responses to different opposition rankings. Greater TD, HSR, and DEC against high-ranked opponents suggest an increased workload in both offensive and defensive aspects of play [29, 30, 33, 34]. The lower Spr against low-ranked opponents may indicate a more controlled and strategic approach, with less emphasis on explosive bursts of speed while attacking and defending.

Attacking midfielders exhibited an intriguing pattern, covering greater TD and performing more DEC against high-ranked opponents. This may signify a strategic focus on ball retention, creativity, and controlled movements against stronger opposition [34]. The lower Spr against high-ranked teams compared to middle-ranked opponents suggest a balance between dynamic play and tactical awareness.

For centre forwards, the greater TD covered against high-ranked opponents aligns with the expectation that this position requires a continued attacking focus but a greater contribution to defensive phases, requiring increased involvement in play [33, 34]. This finding highlights the dynamic nature of the centre forward’s role and the adaptability needed against high-ranked opponents.

### English Premier League

The relationships between physical performance and the quality of opposition in the EPL are elucidated through the analysis of matches played against high-, middle-, and low-ranked opponents. These results offer valuable insights into the physiological demands and strategic adaptations that teams employ in response to varying levels of competition and the demands placed on differing positions.

The observed increase in TD covered in matches against high-ranked opponents resonates with previous research highlighting the heightened physical demands associated with facing stronger competition [31, 35]. This finding suggests that teams engage in a more extensive work rate against high-ranked opponents, emphasizing the significance of endurance and overall physical conditioning in navigating the challenges posed by elite opposition.

The greater HSR and Spr recorded in matches against high-ranked teams provide further granularity to the physical demands placed on players in these competitive scenarios. This heightened intensity may be attributed to the dynamic nature of high-ranked opponents’ playing styles, where rapid transitions and explosive high-intensity bursts and sprinting are integral components [3]. The observed increase in both HSR and Spr highlights the multifaceted nature of elite soccer, requiring players to not only cover vast distances but also exhibit explosive actions such as ACC and DEC both in and out of possession.

The distinct patterns in the number of ACC and DEC emphasizes the tactical adjustments made by teams against different levels of opposition. Matches against high-ranked opponents revealed a higher number of both ACC and DEC, suggesting a more dynamic and physical demanding style of play [2, 3, 12, 29, 31]. The increased number of ACC against high-ranked opponents possibly implies a more defensive approach, potentially involving quick bursts of speed to exploit tactical opportunities or disrupt the opponent’s tactical strategies. Concurrently, the elevated number of DEC notes the need for rapid adjustments in pace or direction, reflecting a greater focus on out of possession principles and the ability to repeatedly press and the intense nature of matches against high-ranked teams. Once again, contrasting results were found in a recent study conducted in English Premier Development League (U-23) [12] in which higher values were found against high-ranked teams.

Regarding playing positions, centre-backs and full-backs displayed no significant differences across any of the examined variables when facing opponents of different rankings. This lack of distinction may highlight the versatility required from defensive players, irrespective of the opponents ranking. Potentially, it suggests that defensive roles demand consistent physical efforts and tactical adaptations, with the absence of significant variations highlighting the adaptability of these positions in responding to different levels of opposition [2, 3, 12, 29–33].

In contrast, centre midfielders exhibited notable variations in physical performance based on opposition rankings. Greater distances covered against high-ranked opponents suggest an increased work rate in the central areas of the pitch when facing strong opposition [2, 3, 33, 34]. Additionally, the observed lower HSR against low-ranked opponents indicates a potentially more controlled and strategic style of play, with less emphasis on explosive bursts of speed. The higher number of DEC against high-ranked opponents suggests a need for frequent adjustments in pace or direction, highlighting the tactical adaptability of centre midfielders in matches against high-ranked teams.

Attacking midfielders displayed distinct patterns, covering greater TD and performing a higher number of DEC when facing high-ranked opponents. This finding aligns with the expectations for players in advanced positions, emphasizing both offensive contributions and the ability to navigate through densely packed defences. The increased number of DEC further indicates a tactical nuance, suggesting a focus on precise movements and creative play against stronger opposition.

For centre forwards, the primary goal-scoring focal point, covered greater TD when facing high-ranked opponents compared to both middle- and low-ranked opponents. This outcome suggests that centre forwards actively contribute not only to the attacking phases but also engage in defensive efforts against high-ranked teams. The observed differences emphasize the dynamic nature of the centre forward’s role and the adaptability required to meet the challenges posed by high-ranked opponents [33–35].

### Practical Applications

Understanding position-specific responses to different opposition strengths can inform targeted training strategies. Coaches may tailor position-specific drills to enhance the physical attributes and decision-making skills that are crucial in specific match scenarios. Moreover, the insights gained from this study may aid in the development of position-specific tactics to exploit opponent weaknesses or mitigate threats based on opposition ranking.

Coaches and sports scientists can leverage these insights to tailor training regimens that simulate the physical and tactical demands associated with different levels of opposition. Position-specific drills and conditioning programs may be designed to enhance players’ capacity for both sustained efforts and explosive actions, catering to the diverse challenges posed by elite competition [11]. Specifically, coaches and performance staff can use the higher values identified in this study to provide additional training as a preparation strategy for the worst-case scenario or the most intense period of match-play considering the different contextual factors such as the level of opponents and playing position [36].

### Limitations and Future Research Direction

While this study highlights relationships between physical performance and opposition rankings, it is essential to acknowledge certain limitations. First, this study included a convenience sample of just one club which limits the generalization of results when describing the results of the two leagues. Moreover, factors such as individual player characteristics, opposition team strategies, and match location may influence these patterns and thus must be considered additional limitations of this study. Future research could therefore consider: (1) individual player profiles and other contextual factors to provide a more comprehensive understanding of the intricacies involved in player performance against varying levels of opposition and identify any further positional differences; (2) exploring the temporal dynamics of these physical performance metrics within individual matches, shedding light on how teams adapt to varying playing styles during different phases of the match against opponents of differing quality; (3) analyzing the weekly training load that precedes match-play to understand if this load alters based on the level of the next opponent as recently highlighted [30]. Finally, it was not possible to discuss the final league position of the analyzed team due to ethical reasons and anonymization for the soccer club and thus should be considered in future studies.

## CONCLUSIONS

In conclusion, there were higher external load values when playing against high- and middle-ranked teams than low-ranked teams. Additionally, the position-specific analyzes of physical performance metrics against high-, middle-, and low-ranked opponents highlight the diverse demands placed on players in elite European soccer. While higher TD was found across all positions when playing against high-ranked teams, all other variables showed distinct patterns for both the ECL and EPL. Regarding centre-backs, this position only differed in the ECL against high-ranked teams showing the lowest HSR distances. Similarly, full-backs only differed in the ECL against both high- and middle-ranked teams performing a higher number of DEC. Centre midfielders also showed identical results in the ECL. However, centre midfielders in the EPL showed the greatest HSR distances and number of DEC against both high- and middle-ranked teams. Attacking midfielders in the EPL showed the lowest Spr distance and the highest number of DEC against high-ranked teams. In the ECL, attacking midfielders again showed the highest DEC against high-ranked teams.

These findings contribute to a more comprehensive understanding of how players from different positions perform in elite soccer match-play against varying opposition rankings. Thus, providing valuable practical insights for coaches and performance staff seeking to optimize training and recovery and tactical approaches based on positional roles and opposition ranking to enhance match outcome. Coaches may then tailor positional and individualized training regimens to address the specific physical demands associated with matches against different-ranked opponents by using the most intense external load values as a strategy for worst-case scenario preparation.
